# Absorption Measurements of Periodically Poled Potassium Titanyl Phosphate (PPKTP) at 775 nm and 1550 nm

**DOI:** 10.3390/s130100565

**Published:** 2013-01-04

**Authors:** Jessica Steinlechner, Stefan Ast, Christoph Krüger, Amrit Pal Singh, Tobias Eberle, Vitus Händchen, Roman Schnabel

**Affiliations:** Institut für Gravitationsphysik, Leibniz Universität Hannover and Max-Planck-Institut fur Gravitationsphysik (Albert-Einstein-Institut), Callinstr. 38, 30167 Hannover, Germany

**Keywords:** PPKTP, absorption, photo-thermal

## Abstract

The efficient generation of second-harmonic light and squeezed light requires non-linear crystals that have low absorption at the fundamental and harmonic wavelengths. In this work the photo-thermal self-phase modulation technique is exploited to measure the absorption coefficient of periodically poled potassium titanyl phosphate (PPKTP) at 1,550 nm and 775 nm. The measurement results are (84±40) ppm/cm and (127±24) ppm/cm, respectively. We conclude that the performance of state-of-the-art frequency doubling and squeezed light generation in PPKTP is not limited by absorption.

## 1. Introduction

There is a multitude of applications for squeezed light. Examples are fundamental research in quantum physics [1–3], the realization of quantum information protocols [4,5], and the sensitivity improvement of gravitational wave detectors (GWDs) [6]. In 2010, a squeezed light laser operating at a wavelength of 1,064 nm was implemented in GEO 600 [7]. Design studies of future generations of GWDs consider the application of strongly squeezed light at 1,064 nm and 1,550 nm [8]. State-of-the-art setups for squeezed light generation require second-order nonlinear crystals that have low loss at the fundamental (squeezed) wavelength as well as at the second-harmonic wavelength that pumps the parametric squeezing process. The non-linear medium is placed inside a singly or doubly resonant optical cavity [9] defining the so-called squeezing resonator. Typical non-linear media are magnesium doped lithium niobate (MgO:LiNbO_3_) [10] and periodically poled potassium titanyl phosphate (PPKTP) [7,11]. The squeezing factor increases with the second harmonic pump intensity; however, it is ultimately limited by optical losses, which include the quantum efficiency of the photo-electric detector, propagation loss, as well as the escape efficiency of the squeezing resonator [12]. To give an example, a total optical loss of 10 % limits the nonclassical noise suppression to a maximum of 10 dB below the shot noise variance. The highest squeezing level demonstrated so far is 12.7 dB at a wavelength of 1,064 nm [11]. The squeezing resonator's escape efficiency is given by [12]

(1) $$\eta =\frac{T}{T+L_{\text{RT}}}$$

where *T* is the transmission of the resonator's coupling mirror and *L*_RT_ is the resonator's round-trip loss with contributions from non-perfect intra-cavity anti-reflection (AR) coatings, from general coating absorption and scattering, and from the bulk absorption of the non-linear crystal. Since a reduction of *L*_RT_ allows a reduction of *T* and thus a reduction of the external pump intensity, the availability of non-linear materials with low absorption is of high interest for the efficient generation of strongly squeezed light. The efficient generation of second-harmonic light sets the same requirement to non-linear materials. The highest efficiency for frequency doubling achieved so far is 95% for continuous-wave light at 1,550 nm [13]. In this work we report on the measurements of the absorption coefficient of PPKTP at 775 nm and at 1,550 nm exploiting the photo-thermal self-phase modulation technique [14].

## 2. Experimental Setups

**Figure 1.**
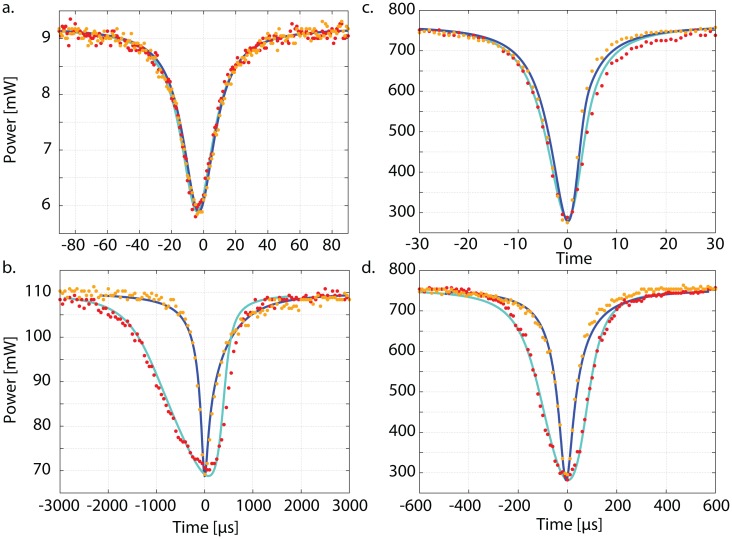
Measured and simulated cavity resonance peaks for an external lengthening (dark-blue line and orange dots) and external shortening (light-blue line and red dots) of the cavity (dots: measurements, lines: corresponding simulations). **a** and **b** were measured with a Fabry–Perot cavity setup at 775 nm (cavity line-width ≈ 10 MHz) using an input power of 9mW at a scan frequency of 550 Hz (**a**) and 110mW at 15Hz (**b**). **c** and **d** were measured with a bow-tie cavity setup at 1,550 nm (cavity line-width ≈ 750 kHz) at an input power of 760mW at scan frequencies of 149Hz (**c**) and 11Hz (**d**). For low power and a high scan frequency, no thermal effect occurs (**a**). For slower scan frequencies and higher powers, the narrow peaks form for an external lengthening and the broad peaks for an external shortening of the cavity (**b**–**d**). From those measurements we derived the absorption coefficients as summarized in Figure 2.

The photo-thermal self-phase modulation technique is an absorption measurement method that exploits the deformation of cavity resonance peaks due to absorption in the intra-cavity bulk material [14] or in the coating of the cavity's in-coupling mirror [15]. The resonance peaks are observed while changing the cavity length with a piezo-electric transducer (PZT) attached to one of the cavity mirrors. For zero absorption the resonance peaks are identical for external shortening and lengthening. The situation changes when absorption takes place. Then, for most materials, heating leads to thermal expansion and to a change of the refractive index. In the case of PPKTP, the cavity round-trip length increases as soon as the intra-cavity light power builds up. For an external lengthening, the resonance peak becomes narrower, and for an external shortening, the resonance peak becomes broader. Figure 1 shows four example measurements of resonance peaks detected in reflection of a Fabry–Perot (a and b) and a bow-tie cavity (c and d) and the corresponding simulations. Shown are the peaks with no thermal effect (a), low thermal effect (c) and a clearly visible thermal effect (b and d). While the peaks in Figure 1a show no thermal effect, they are identical for both scan directions. For peaks with thermal effect, an external shortening of the cavity forms broad peaks (simulation: light-blue line, measurement: red dots) and an external lengthening of the cavity forms narrow peaks (simulation: orange line, measurement: dark-blue dots). The thermal effect increases with increasing input laser power and vanishes for very low power. Changing the scan velocity also has an effect since faster scanning results in less deposited energy. As the scan velocity increases, the thermal effect decreases. Material parameters such as the heat conductivity also need to be considered to quantitatively describe the deformation of the resonance peak.

Solid lines in Figure 1 correspond to our quantitative simulation using the parameters from Table 1 as well as the absorption coefficient *α* as fitting parameters. The simulation also requires the cavity mirror reflectivities *R*_1_ and *R̃*_2_, which are also determined from these measurements. As a consistency check *R*_1_ and *R̃*_2_ additionally were determined in independent measurements, which show no thermal effect. *R̃*_2_ is the *effective* reflectivity of the end-mirror. It includes all cavity round-trip losses apart from the transmission of the in-coupling mirror. The values for the material parameters were taken from literature [16–19]. The time axis of the measurements is calibrated using frequency markers in terms of phase-modulation sidebands generated by an electro-optical modulator. These sidebands are slightly outside the window shown here. Using frequency markers for each measurement also minimizes errors caused by the hysteresis of the PZT. A set of fitting parameters is then found by minimizing the standard deviation between the measurement data and the simulation employing a Nelder–Mead algorithm. To minimize errors due to transient disturbances, multiple measurements of resonance peaks were performed. Also, the scanning speed as well as the light power was varied, providing a self-consistency check of our evaluation.

In the following sections the experimental setups for the absorption measurements at both wavelengths and the obtained results are presented. For the absorption measurements existing SHG and squeezing cavities were used, which resulted in different cavity types and simultaneously demonstrates the manifold application possibilities of the measurement method.

For the 775 nm absorption measurement we used a half-monolithic standing wave cavity, while for the 1,550 nm absorption measurement a traveling-wave bow-tie cavity was used. In a standing wave setup, the absorption depends on the position of the substrate relative to the emerging node-antinode pattern [20]. However, this effect is negligible for our measurements, since the substrate dimensions are much larger than the wavelength *λ*. Traveling-wave cavities generally have the advantage that the reflected beam is easily accessible for detection, since it does not coincide with the incoming beam. Another advantage is that disturbances due to parasitic cavities between the anti-reflection coating and the high reflection coating of the coupling mirror are less likely. Standing wave cavities on the other hand can be realized in a more compact way minimizing acoustic vibrations. In the work presented here, however, both cavity types turned out to be suitable for absorption measurements.

### 2.1. Experimental Setup and Results at 775 nm

For the absorption measurements at a wavelength of *λ* = 775 nm we used a half-monolithic standing-wave cavity. Figure 3a shows the schematic of our experiment. The beam splitter (BS) reflected most of the light towards the in-coupling mirror M_1_. The PPKTP crystal was manufactured by Raicol [21] and had a curved end surface with a high reflection (HR) coating applied to it. The other end surface was plane and AR coated. The cavity was formed by the in-coupling mirror *M*_1_, which was placed 24 mm in front of the AR coating of the crystal and the crystal's HR coating. A photo diode (PD) detected the light power P_refl_(t), which was reflected from the in-coupling mirror M_1_ and partly transmitted by the BS. The input light carried phase modulation sidebands imprinted by an EOM. The modulation frequency was chosen to be 101.25 MHz, which is outside the cavity line width of ≈11 MHz, so the crystal could cool down before the sidebands became resonant. The PD's photo current was demodulated at the EOM modulation frequency by means of a double-balanced mixer to generate frequency markers in terms of a Pound Drever Hall (PDH) error signal [22]. This way, we were able to precisely calibrate the motion of the piezo-driven mirror M_1_ around the cavity resonance.

**Figure 2.**
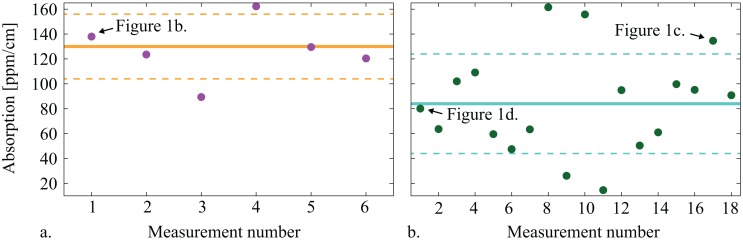
The dots show the absorption coefficient obtained from individual measurements at 775 nm (left) and at 1,550 nm (right). The mean value (line) and standard deviation (dashed lines) of the absorption coefficient are *α*_775nm_ = 127 ± 24 ppm/cm and *α*_1,550nm_ = 84 ± 40 ppm/cm. The absorption results corresponding to the peaks shown in Figure 1 are labeled.

Altogether we performed 13 individual measurements using three different input powers of 9mW, 37mW and 110mW. The scan frequencies were varied by almost a factor of a hundred. Six of the 13 measurements showed a strong thermal effect and permitted to obtain an absorption coefficient as well as the reflections of *R*_1_ and *R̃*_2_. From the remaining 7 measurements only *R*_1_ and *R̃*_2_ were independently determined and were found to be in agreement with the first 6 measurements. Figure 2 shows the results of the individual measurements of the absorption coefficient *α* (purple dots, right graph) as well as their mean value (thick yellow line) and their standard deviation (dashed yellow lines) of *α*_775nm_ = (127 ± 24) ppm/cm. The result for the power reflectivity of M_1_ was R_1_ = (98.33 ± 0.08)%, which agreed with the specified design value of R_1_ = (98.5 ± 0.4)%. The result for the effective reflectivity was *R̃*_2_ = (99.76 ± 0.01)%. The designed reflectivity for the HR coating was about R_2_ = 99.95%. The residual loss of about 1,900 ppm per round-trip due to absorption, scattering and reflection at the AR-coating is compatible with the specification of the AR coating of *R* < 0.1 %.

The design reflectivity *R*_1_ of the in-coupling mirror of this setup at 1,550 nm was 90%, while the end-surface of the crystal was HR coated. Due to this strong impedance mismatch of the mirror reflectivities, only ≈ 7% of the laser power are transmitted into the cavity at 1,550 nm at resonance instead of ≈ 30% at 775 nm. The resolution of the peaks at 1,550 nm becomes inferior compared with 775 nm and therefore the measured peaks become noisier. Hence, a setup with more suitable parameters was used for the measurement at 1,550 nm.

### 2.2. Experimental Setup and Results at 1,550 nm

**Figure 3.**
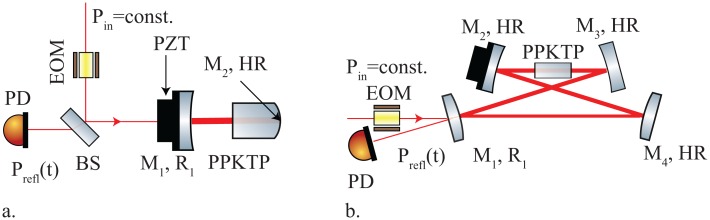
(**a**) half-monolithic cavity setup for the absorption measurement at 775 nm: Mirror M_1_ and the HR coating on the PPKTP crystal's curved end surface formed the cavity. The length of the cavity was scanned with the PZT onto which M_1_ was mounted. A photo diode (PD) detected resonance peaks P_refl_(t) in reflection of M_1_; (**b**) Bow-tie cavity setup for the absorption measurement at 1,550 nm: The in-coupling mirror M_1_ and three HR-coated mirrors formed a bow-tie ring-cavity. The PPKTP crystal was placed within the small waist between the concave mirrors M_2_ and M_3_. M_2_ was moved by a PZT. The PD detected the resonance peak P_refl_(t) in reflection of M_1_. In both setups the beam passed an EOM for imprinting sidebands before entering the cavity for the calibration of the mirror motion.

The absorption measurement at a wavelength of 1,550 nm was performed in a four mirror bow-tie ring-cavity setup. Figure 3b shows a schematic of the experiment. Mirror M_1_, which had a design power reflection of 99%, couples the light into the cavity. The remaining three mirrors were HR coated. While M_1_ and M_4_ were convex, M_2_ and M_3_ were concave forming a waist of 30.5 *μ*m that was located in the centre of the PPKTP crystal. The latter was again produced by Raicol [21]. Both crystal surfaces were AR-coated. The optical round-trip length of the cavity was 832 mm.

The measurement was performed using p-polarized light to avoid the phase matching condition for second-harmonic generation. The p-polarization also had the advantage that the high reflective cavity mirrors showed a slightly reduced reflectivity, which improved the signal-to-noise ratio of our measurement due to a reduced impedance mismatch. The detection of the resonance peaks and the frequency markers was identical to the procedure described in Subsection 2.1.

From this setup, 18 individual measurements were recorded. We used a constant input power of 760 mW but varied the scan frequency from 11 Hz up to 200 Hz. The measurements at 149 Hz and 11 Hz are shown in Figure 1c,d.

The time axis of Figure 1d shows that the scan frequency of 11 Hz corresponded to a scanning time of about 200 *μ*s per resonance peak at full width half maximum (FWHM). The scan amplitude was not changed within the series of measurements so the scanning time per FWHM changed in proportion to the scan frequency. All 18 measurements showed a thermal effect and allowed to obtain an absorption coefficient besides *R*_1_ and *R̃*_2_.

The mean value and standard deviation for the absorption coefficient were *α*_1,550nm_ = (84 ± 40) ppm/cm. This non-monolithic setup with a very large round-trip length in combination with the small waist is very susceptible to acoustic and thermal fluctuations, which most likely caused the large error bar. The result for the reflection of M_1_ was *R*_1_ = (99.03 ± 0.10)%, which agrees with the design value of 99%. The result for the effective reflection was *R̃*_2_ = (99.76 ± 0.04)%. *R̃*_2_ again included all cavity round-trip losses, which are the absorption of the PPKTP crystal, the reflection of the two AR coatings, the transmission of the three HR mirror coatings as well as the absorption and scattering of all four mirrors. A loss of 1 − *R̃*_2_ ≈ 4,000 ppm per round trip is a reasonable result for p-polarized light.

Our bow-tie cavity is usually operated with s-polarized light at 775 nm as well as at 1,550 nm for which the PPKTP crystal is quasi-phase-matched. To be able to compare the crystal's absorption with other cavity round trip losses for s-polarized light, we performed another series of eight individual loss measurements. The polarization was rotated to s-pol and the light power was strongly reduced so that no thermal deformation occurred. The crystal's temperature was tuned far away from the quasi-phase-matching regime to avoid SHG. The result for the effective reflection is *R̃*_2_ = (99.935 ± 0.008)%, which corresponds to a round-trip loss of (650 ± 80) ppm. Thus, for our cavity operated in s-pol, the absorption of the crystal of 84 ppm/cm (crystal length: 1 cm) is not the dominating loss source.

## 3. Conclusions

In this work, the absorption coefficient of PPKTP [21] was measured at the quasi-phase matched wavelengths of 775 nm and 1,550 nm using the photo-thermal self-phase modulation technique. We used two almost identical crystals of 10 mm and 9.3 mm length in two geometrically different cavity setups, a half-monolithic standing-wave cavity and a traveling-wave bow-tie cavity. The results for the absorption coefficients are *α*_775nm_ = (127 ± 24) ppm/cm and *α*_1550nm_ = (84 ± 40) ppm/cm. The error bars correspond to one standard deviation excluding systematic effects due to errors in the material parameters. The latter are, however, estimated to be smaller than our statistical error bars. If such a crystal is used in an otherwise lossless squeezing resonator with an in-coupling reflectivity of *R* ≈ 90%, the escape efficiency would be as high as *η* > 99.9%. With this value, a loss of merely 1% is associated, thus allowing the generation and observation of squeezing strengths and second harmonic conversion efficiencies beyond what has been achieved so far. The highest squeezing factor at 1,550 nm observed so far is 12.3 dB [23] with a total optical loss of the setup of 3.5%. The highest efficiency for external continuous-wave second harmonic generation so far is 95% [13]. We conclude that the bulk absorption of PPKTP, by far, does not limit state-of-the-art squeezed light and second-harmonic generation at this wavelength.

## Figures and Tables

**Table 1.** Material and geometric parameters of the bow-tie cavity and the half-monolithic cavity used for the simulations.

**Geometric parameters**	**775nm**	**1,550 nm**	**References**
Beam waist *ω*_0_	27.6 *μ*m	30.2 *μ*m	
Crystal length *L*	9.3mm	10mm	
Crystal radius *R*	1.5mm	1.5mm	
Air gap	24mm	832mm	
**Material parameters**			

Index of refraction *n*	1.85	1.82	[16]
Thermal refr. coeff d*n*/d*T*	16.9 × 10^−6^/K	10.9 × 10^−6^/K	[17]
Specific heat *c*	726 J/(kgK)		[18]
Density *ρ*_KTP_	2, 945 kg/m^3^		[19]
Thermal expansion *a*_th_	0.6 × 10^−6^/K		[18]
Thermal conductivity *k*_th_	2.23 W/(mK)		[18]
Material emissivity *ϵ*	1.0^a^		

a 0.0 < *ϵ* ≤ 1.0 are the boundaries for the thermal emissivity. For our systems the value of this parameter is not relevant since *R* ≫ *ω*_0_ and therefore surface radiation is negligible.
